# A Novel Antibacterial Component and the Mechanisms of an *Amaranthus tricolor* Leaf Ethyl Acetate Extract against *Acidovorax avenae* subsp. *citrulli*

**DOI:** 10.3390/ijms23010312

**Published:** 2021-12-28

**Authors:** Ya Zhang, Ke Gao, Chong Wang, Shuangqing Liu

**Affiliations:** 1 College of Plant Protection, Hunan Agricultural University, Changsha 410128, China; zhangya230@126.com (Y.Z.); gaoke@stu.hunau.edu.cn (K.G.); 2 College of Bioscience and Biotechnology, Hunan Agricultural University, Changsha 410128, China

**Keywords:** *Amaranthus tricolor*, bacteriostatic component, identification, ultrastructure observation, metabolome, enzymatic activity, molecular docking

## Abstract

The aim of the present investigation was to determine the active ingredients in *Amaranthus tricolor* L. leaves and develop a biological pesticide. Organic solvent extraction, column chromatography, liquid chromatography, ODS-C18 reverse elution, Sephadex LH-20 gel filtration, H spectrum, and C spectrum were used to isolate the pure product for an assessment of the agricultural activity and bacteriostatic mechanisms. The results showed that the activity of the crude extract following carbon powder filtration was 1.63-fold that of the non-filtered extract. Further isolation was performed to obtain two pure products, namely, hydroxybenzoic acid (HBA) and benzo[b]furan-2-carboxaldehyde (BFC), and their molecular formulas and molecular weights were C_7_H_6_O_3_ and 138.12, and C_9_H_6_O_2_ and 146.12, respectively. Our study is the first to determine that HBA has bacteriostatic activity (MIC 125 μg/mL) and is also the first to isolate BFC from *A. tricolor*. The ultrastructure observation results showed that HBA caused the bacteria to become shriveled, distorted, and deformed, as well as exhibit uneven surfaces. After HBA treatment, 70 differentially expressed metabolites were detected in the bacteria, of which 9 were downregulated and 61 were upregulated. The differentially expressed metabolites were mainly strigolactones, organic acids and derivatives, fatty acids, benzene and substituted benzene derivatives, amino acids and associated metabolites, and alcohols and amines. Among all of the downregulated differentially expressed metabolites, MEDP1280 was the most critical, as it participates in many physiological and biochemical processes. The enrichment analysis showed that the differentially expressed metabolites mainly participate in tyrosine metabolism, biosynthesis of amino acids, cysteine and methionine metabolism, and arginine and proline metabolism. Additionally, HBA was found to disrupt cell membrane permeability and integrity, causing the leakage of substances and apoptosis. The physiological and biochemical test results showed that HBA could increase the pyruvate levels in bacteria but could decrease the activities of respiratory enzymes (malate dehydrogenase (MDH) and NADH oxidase) and antioxidant enzymes (superoxide dismutase (SOD) and glutathione peroxidase (GSH-PX)). Inverse molecular docking was used to study the binding between HBA and respiratory and antioxidant enzymes. The results showed that HBA could bind to MDH, NADH oxidase, SOD, and GSH-PX, suggesting that these enzymes may be the effector targets of HBA. Conclusion: The optimal active ingredient in *A. tricolor* that can inhibit *Acidovorax avenae* subsp. *citrulli* was identified as HBA. HBA mainly disrupts the cell membrane, damages the metabolic system, and inhibits respiration and antioxidant enzyme activity to control bacterial growth. These results provide a reference for the further development of biological pesticides.

## 1. Introduction

*Amaranthus tricolor* L. is a dicotyledonous herb that is widely cultivated in tropical and temperate regions [1]. It exhibits strong stress resistance, including drought and barren land resistance, grows easily, requires little time investment for agricultural production, and has a high survival rate [2,3]. It also has high nutritional value, being rich in vitamins and minerals, and is mainly consumed as a leafy vegetable [4,5]. Additionally, *A. tricolor* has a high red pigment content, and its pigments are typically extracted industrially to prepare food additives [6]. A study by Yustiati et al. showed that *A. tricolor* can be used as an animal feed to increase production efficiency in animals [7]. In addition, many studies have found that amaranth plants have medicinal value, and *A. tricolor* exhibits in vivo anti-cancer activity. Jayaprakasam et al. showed that *A. tricolor* has exceptionally good tumor control results [8], and *A. tricolor* has been found to decrease glucose and labor pain. Rahmatullah et al. found that *A. tricolor* can be used as an antihyperglycemic and antinociceptive drug [9]. Other studies have also demonstrated that *A. tricolor* has antioxidant effects. For instance, Khandaker (2008) and Alam (2013) found that *A. tricolor* can be used as an antioxidant to treat refractory diseases [10,11]. Khanam et al. found that the phenol content of *A. tricolor* is intimately associated with its antioxidant capacity [12]. Another study also found that *A. tricolor* has hepatoprotective effects. Al-Dosari et al. showed that *A. tricolor* extract can decrease liver disease-related markers such as glutamate oxaloacetate transaminase (GOT), alkaline phosphatase (ALP), bilirubin, and cholesterol, and it can be used as a natural hepatoprotectant for treating liver diseases [13]. *Amaranthus tricolor* can also prevent obesity, of which 50 µg/mL EERS showed strong prophylactic effects in diet-induced obesity in zebrafish [14]. Many studies have shown that *Amaranthus* plants have good inhibitory effects on contaminating bacteria and fungi in foodstuffs. Pulipati et al. found that the methanol extract of *A. tricolor* exhibited the best inhibitory effect on *Escherichia coli*, followed by ethyl acetate, chloroform, and petroleum ether extracts [15]. Oh et al. [16] found that the chloroform extract of *Amaranthus lividus* has high inhibitory effects on foodborne bacteria, such as *Listeria monocytogenes*, *Salmonella* spp., and *Staphylococcus aureus*. AbdAziz et al. [17] also found that the active ingredients of *Amaranthus mangostanus* have strong inhibitory effects on common foodborne bacteria. Lina [18] and Lyapkova [19] discovered that *Amaranthus hypochondriacus* and *Amaranthus caudatus* contain many microbiostatic substances that act against saprophytic fungi. However, there are very few studies on the agricultural value of *A. tricolor*, and its potential active substances remain to be discovered and utilized. As there are differences in the pathogenesis between foodborne bacterial and fungal pathogens, and agricultural bacterial and fungal pathogens, we wondered whether *A. tricolor* also exhibits inhibitory effects on agricultural bacterial and fungal pathogens, as it does show inhibitory effects on foodborne bacterial and fungal pathogens. Studies have found that *A. tricolor* exerts numerous inhibitory effects on many plant pathogens, but it is not clear what the active substances are. It is also unclear whether this substance is a novel compound or a known compound or whether it has a novel structure. This limits the development and application of *A. tricolor* [20]. To address this, *Acidovorax avenae* subsp. *citrulli*, a plant quarantine bacterium, was used as an indicator bacterium on an ethyl acetate leaf extract of *A. tricolor*. The extract was filtered with activated carbon to remove pigment prior to column chromatography. Thin-layer chromatography (TLC), ODS-C18 reverse elution, Sephadex LH-20 gel filtration, and nuclear magnetic resonance (NMR) were used for isolation and purification, followed by structure identification. Ultrastructure observation, metabolomics analysis, physiological and biochemical tests, and molecular docking techniques were used to study the effector mechanisms of critical bacteriostatic molecules in order to discover lead compounds with novel structures as well as determine bacteriostatic molecular targets to provide a theoretical basis for further development of biological pesticides.

## 2. Results

### 2.1. Bacteriostatic Activity of the Activated Carbon Chromatography Eluate of A. tricolor

**Figure 1:**
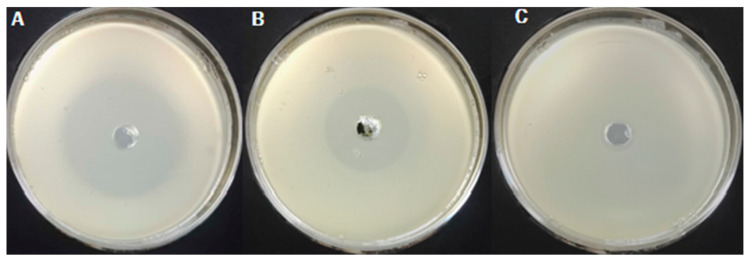
Antibacterial activity of ethyl acetate extract before and after elution by carbon column chromatography. (**A**) Treated by carbon column; (**B**) not treated by carbon column; (**C**) blank control.

As shown in Figure 1, the zone of inhibition of the *A. tricolor* ester extract that underwent activated carbon chromatography and did not undergo activated carbon chromatography was 4.45 and 2.73 cm, respectively. The zone of inhibition of the *A. tricolor* ester extract that underwent activated carbon chromatography was 1.63-fold that of the *A. tricolor* ester extract that did not undergo activated carbon chromatography. This showed that activated carbon chromatography enhanced the inhibitory effects of the *A. tricolor* ester extract on *A. avenae* subsp. *citrulli* and that ‘impurities’ were adsorbed on the activated carbon, and hence, the activated carbon had a purification role toward the extract.

### 2.2. A. tricolor Ester Extract Isolation and Purification

Trichloromethane: methanol (1:0, 9:1, 4:1, 7:3, 3:2, 0:1) was used for gradient elution of the silica gel column. Collection was performed every 250 mL, and 87 elution bottles were collected. After testing, 20 fractions were combined, and activity testing was performed to obtain nine bacteriostatic fractions. An ODS-18 reverse-phase silica gel column was used, and methanol was used for gradient elution. Collection was performed every 100 mL, and 83 elution bottles were collected. After testing and combining, seven fractions were obtained, and activity testing was performed to obtain one bacteriostatic fraction. This bacteriostatic fraction underwent Sephadex LH-20 silica gel column separation, and 100% methanol was used for elution. Collection was performed every 5 mL, and 54 elution bottles were collected. After testing, compound 1 with a purity of 95.68% (52.60 mg) and compound 1 with a purity of 95.73% (33.90 mg) were collected and named compounds 1 and 2.

### 2.3. Structural Identification of Pure Products

**Figure 2:**
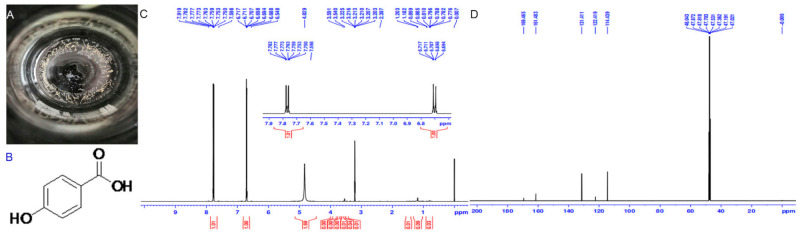
Morphology and structure identification of HBA. Note: (**A**) physical state; (**B**) structural formula; (**C**) ^1^H-NMR diagram of HBA (500 MHz, MeOD); (**D**) ^13^C-NMR of HBA (126 MHz, MeOD).

Compound **1**: yellowish-white needle crystals (methanol), molecular formula was C_7_H_6_O_3_, and molecular weight was 138.12. 1 H NMR (500 MHz, MeOD) δ 7.80–7.73 (m, 82 H), 6.74–6.67 (m, 81 H), 4.83 (s, 135 H), 3.61–3.50 (m, 4 H), 3.21 (dt, J = 3.3, 1.7 Hz, 35 H), 1.18 (s, 7 H), 0.86–0.76 (m, 2 H), 0.03–0.06 (m, 11 H); 13 C NMR (126 MHz, MeOD) δ 170.94 (s), 162.96 (s), 132.89 (s), 123.89 (s), 115.91 (s), 49.52 (s), 49.26 (d, J = 21.4 Hz), 49.01 (s), 48.84 (s), 48.67 (s), 48.50 (s). The above data were consistent with that reported by Chen et al. [21]. Therefore, this compound was identified as 4-hydroxybenzoic acid (HBA) (Figure 2).

**Figure 3:**
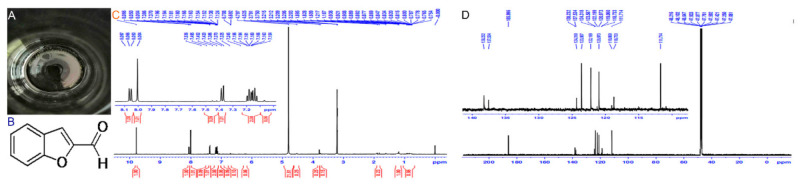
Morphology and structure identification of BFC. Note: (**A**) physical state; (**B**) structural formula; (**C**) ^1^H-NMR diagram of BFC (500 MHz, MeOD); (**D**) ^13^C-NMR of BFC (126 MHz, MeOD).

Compound **2**: yellowish-white powder (methanol), molecular formula was C_9_H_6_O_2_, and molecular weight was 146.14. ^1^H NMR (500 MHz, MeOD) δ7.66 (d, J = 16.2 Hz, 3 H), 6.12 (d, J = 16.1 Hz, 3 H), 5.82 (s, 3 H), 5.66 (s, 3 H), 5.41–5.30 (m, 2 H), 4.83 (s, 94 H), 3.84 (dd, J = 17.2, 9.3 Hz, 2 H), 3.52 (d, J = 26.9 Hz, 4 H), 3.52–3.37 (m, 1 H), 3.21 (dt, J = 3.3, 1.6 Hz, 21 H), 2.62 (t, J = 6.4 Hz, 18 H), 2.51–1.89 (m, 90 H), 1.82 (dd, J = 13.3, 4.7 Hz, 11 H), 1.62 (d, J = 13.3 Hz, 2 H), 1.57–1.34 (m, 50 H), 1.34–1.05 (m, 76 H), 1.07 (d, J = 17.8 Hz, 1 H), 1.11–0.99 (m, 5 H), 0.95 (d, J = 17.9 Hz, 19 H), 0.81 (t, J = 7.0 Hz, 8 H), 0.00 (s, 1 H); ^13^C NMR (126 MHz, MeOD) δ48.45–47.67 (m), 47.60 (s), 47.42 (s), 47.25 (s), 47.08 (s). The above data were consistent with the literature [22]. Therefore, the compound was identified as benzo[b]furan-2-carboxaldehyde (BFC) (Figure 3).

### 2.4. Virulence Measurement of A. tricolor Active Ingredients

**Figure 4:**
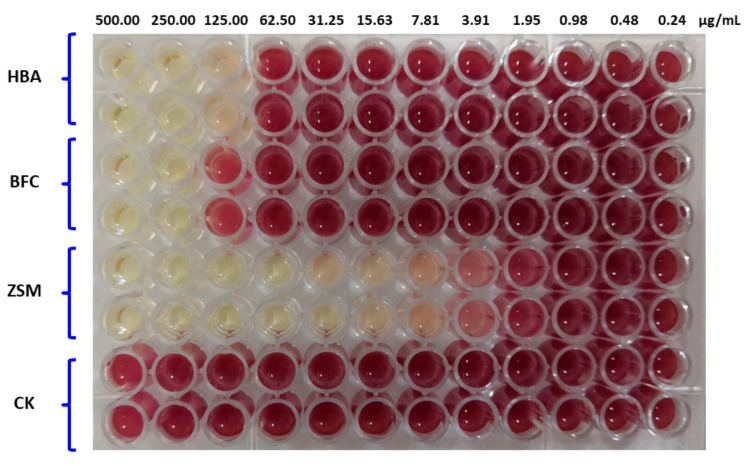
Determination of MIC value of novel fungicide HBA against *A. avenae* subsp. *citrulli*. Note: HBA: 4-hydroxybenzoic acid; BFC: benzo[b]furan-2-carboxaldehyde; ZSM: zhongshengmycin; CK: control.

From Table 1, it can be seen that HBA had inhibitory effects on *A. avenae* subsp. *citrulli*, and the zone of inhibition was 2.29 cm, which was comparable to the positive control and the difference was not significant (*p* > 0.05). BFC did not have any bacteriostatic effects on *A. avenae* subsp. *citrulli*, and the zone of inhibition was lower than the positive control (Table 1). Two-fold dilution in a 96-well plate was used for MIC measurement of HBA. The results indicated that the MIC of HBA towards *A. avenae* subsp. *citrulli* was 125 μg/mL, thus showing potent bacteriostatic activity with potential for further research and development (Figure 4).

### 2.5. Ultrastructure Observation of Bacteria Inhibited by HBA

**Figure 5:**
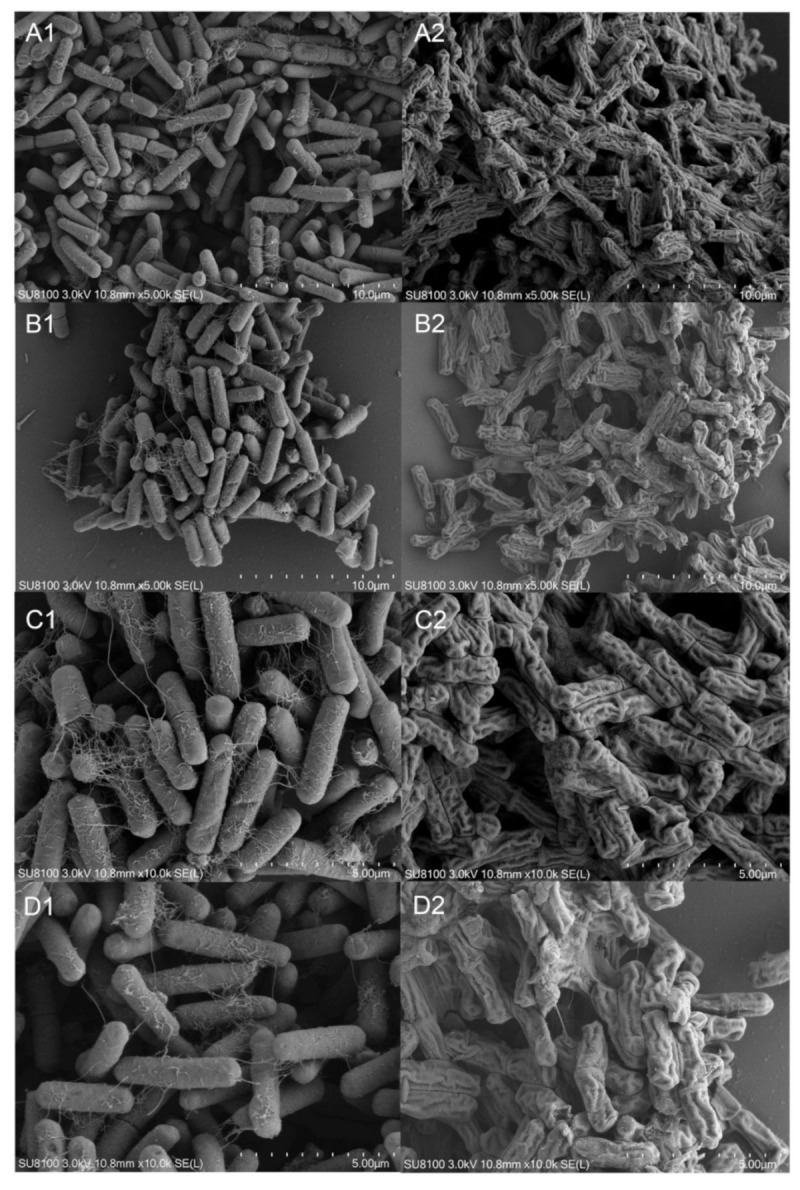
Morphology and structure alterations of HBA-treated *A*. *avenae* subsp. *citrulli* observed by SEM. Note: (**A1**,**B1**): The control *A. avenae* subsp. *citrulli* with 5000 times. (**C1**,**D1**): The control *A. avenae* subsp. *citrulli* with 10,000 times. (**A2**,**B2**): The treated *A. avenae* subsp. *citrulli* with 5000 times (125 μg/mL). (**C2**,**D2**): The treated *A. avenae* subsp. *citrulli* with 10,000 times (125 μg/mL).

Under 5000× magnification, the untreated bacteria appeared rod-shaped, filled, and rounded, with clear edges, neatly stacked and arranged, and consistent in size, and ruptures and distortion were absent (Figure 5A1,B1). The HBA-treated bacteria were shriveled, wrinkled, distorted, and deformed, and the bacterial surface was uneven and even ruptured (Figure 5A2,B2). Under 10,000× magnification, the untreated bacteria had clear edges and smooth surfaces, protrusions and depressions were absent, and many filaments were present (Figure 5C1,D1). The HBA-treated bacteria had a coarse and uneven surface and inconsistent depth, and some bacteria were ruptured or disintegrated (Figure 5C2,D2). This demonstrated that HBA could cause bacterial deformation.

### 2.6. Effects of HBA on the Bacterial Metabolome

**Figure 6:**
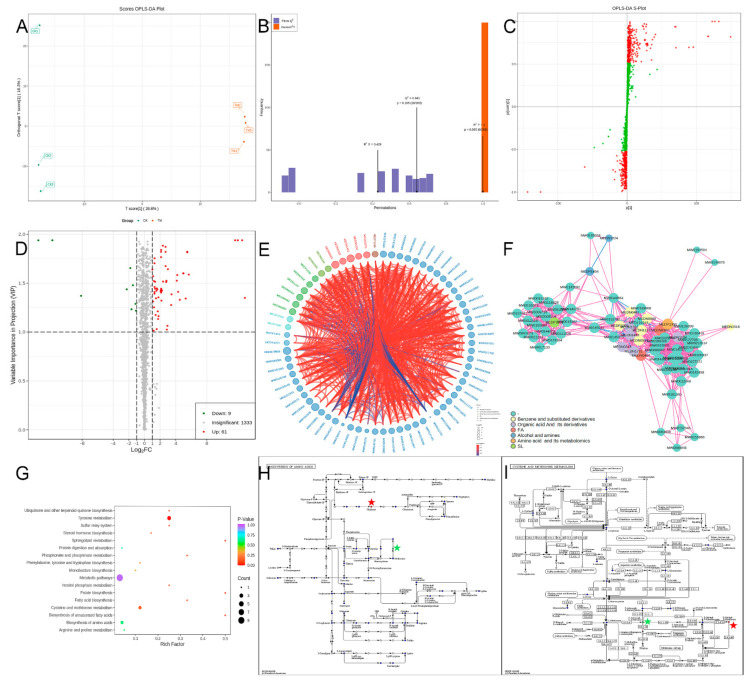
Metabolite’s analysis of HBA-treated *A. avenae* subsp. *citrulli* by LC-MS/MS. Notes: (**A**): OPLS-DA score plot; (**B**): OPLS-DA verification diagram; (**C**): OPLS-DA S-plot; (**D**): difference metabolite volcano map; (**E**): difference metabolites and dazzle diagram; (**F**): difference metabolite network diagram; (**G**): difference metabolites KEGG enrichment; (**H**): biosynthesis of amino acids difference metabolites KEGG pathway; (**I**): cysteine and methionine difference metabolites KEGG pathway.

Orthogonal partial least squares-discriminant analysis (OPLS-DA) removes systemic differences that are unrelated to groups to screen for differentially expressed metabolites and achieve maximum separation. The prediction parameters of the evaluation model include R^2^X, R^2^Y, and Q^2^, of which R^2^X and R^2^Y represent the explanatory power of the constructed model on the X and Y matrices and Q^2^ represents the predictive capacity of the model. The closer these markers are to 1, the more reliable the model. The model is considered to be effective when Q^2^ > 0.5. An S-plot was used to identify differentially expressed compounds related to the groupings. The supervised OPLS-DA was performed on the treatment group and control group. The results showed that the inter-group differences were significant, whereas the intra-group differences were not significant (Figure 6A). From Figure 6B, it can be seen that when OPLS-DA was performed, the OPLS-DA score charts of the treatment group and control group were R^2^X = 0.429, R^2^Y = 1, and Q2 = 0.641, showing that this model has good accuracy and predictive power. The corresponding S-plot shows the most related metabolites in the two groups (Figure 6C).

There were 70 differentially expressed metabolites in the HBA-treated bacteria, of which 9 metabolites were downregulated and 61 metabolites were upregulated (Table 2, Figure 6D). These differentially expressed metabolites may be potential biological targets and include strigolactones, organic acids and derivatives, fatty acids, benzene and substituted benzene derivatives, amino acids and associated metabolites, alcohols and amines, and other metabolites (Figure 6E). The correlation of differentially expressed metabolites was further studied, and the results showed that most metabolites had a close relationship and only a few metabolites did not have a close relationship (Figure 6E). This shows that many metabolites have synergistic or inter-regulatory relationships and play similar or identical roles in life activities. From the network correlation of differentially expressed metabolites, it can be seen that most differentially expressed metabolites had good network correlation, with correlations being present between different metabolites and only a few metabolites exhibiting poor correlations. 3,7,4′-Trihydroxyflavone (MW0132792), ancistrocladine (MW145074), aminopentol (MW0145031), 5′-S-methyl-5′-thioinosine (MW0143701), and 5′-oxoinosine (MW0143692) acted as links in many metabolite relationships, of which 3,7,4′-trihydroxyflavone (MW0132792) downregulation affected many metabolites (Figure 6F). Hence, there are synergistic or inter-regulatory relationships between these metabolites. Differentially expressed metabolites interact with each other to form different pathways. The Kyoto Encyclopedia of Genes and Genomes (KEGG) metabolic pathways are indicated in Figure 6H. Changes in metabolic pathways such as tyrosine metabolism, biosynthesis of amino acids, cysteine and methionine metabolism, arginine and proline metabolism, sulfur relay system, inositol phosphate metabolism, and phosphonate and phosphinate metabolism occurred after the bacteria were treated with HBA, and changes were the greatest in the biosynthesis of amino acids and cysteine and methionine metabolism. Disruption of these metabolic pathways inhibited bacterial growth (Figure 6I). This showed that HBA could alter metabolic pathways to inhibit bacterial growth and control bacteria. In-depth analysis of the metabolite changes in metabolic pathways that were greatly affected (tyrosine metabolism, biosynthesis of amino acids, cysteine and methionine metabolism) by HBA showed that 2,5-dihydroxy benzoic acid (MEDN0089), phenol (MEDL01916), and 2-(4-Hydroxyphenyl) ethanol (MEDN0554) in tyrosine metabolism were upregulated, which may be related to drug resistance. S-Adenosyl-L-methionine (MEDP1280) in the biosynthesis of amino acids was downregulated, which may be an HBA target, and S-adenosyl-L-methionine (MEDP1280) in cysteine and methionine metabolism was also downregulated, whereas MW0143701 was regulated, which may be related to HBA resistance. Obviously, S-adenosyl-L-methionine (MEDP1280) was also downregulated in other metabolic pathways, showing that this substance plays important roles in many metabolic pathways.

### 2.7. Effects of HBA on Bacterial Enzymatic Activity

**Figure 7:**
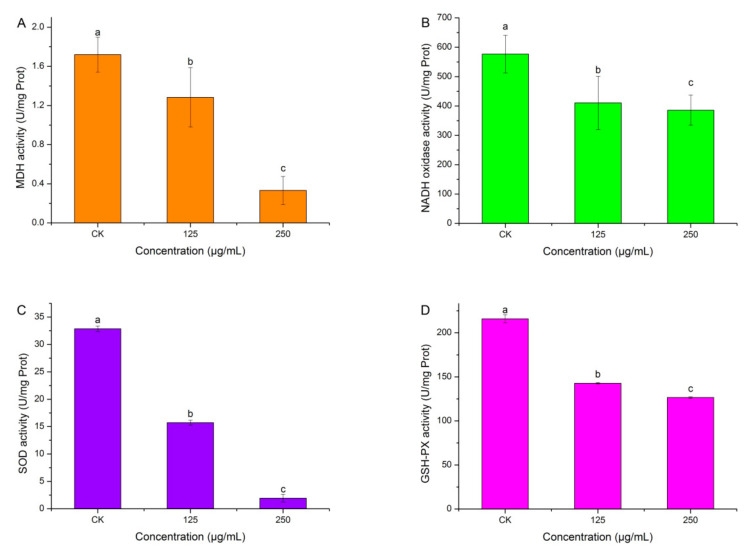
Effect of HBA against respiratory enzymes and antioxidant enzymes activity. Note: (**A**): MDA: malate dehydrogenase; (**B**): NADH: NADH oxidase; (**C**): SOD: total superoxide dismutase; (**D**): GSH-PX: glutathione peroxidase. Columns followed by different letters indicate significantly different scores in the same phase according to Duncan’s multiple range tests at the *p* < 0.05 significance level.

Malate dehydrogenase is a key enzyme in bacterial respiration. Figure 7A shows that HBA could inhibit malate dehydrogenase activity. HBA concentration was negatively correlated with malate dehydrogenase activity. When the HBA concentration was 250 μg/mL, malate dehydrogenase activity was the lowest at 0.33 U/mg Prot, which was 0.19-fold that of the control (*p* < 0.05). This shows that HBA could inhibit bacterial respiration, resulting in decreased energy and weak metabolism, thereby inhibiting bacterial growth.

NADH oxidase is an important enzyme in bacterial respiration and is also an important antioxidant enzyme. Figure 7B shows that HBA could inhibit NADH oxidase activity. HBA concentration was negatively correlated with NADH oxidase activity. When the HBA concentration was 250 μg/mL, NADH oxidase activity was the lowest at 385.83 U/mg Prot, which was 0.67-fold that of the control (*p* < 0.05). This shows that HBA could inhibit bacterial respiration, resulting in decreased energy and weak metabolism, thereby inhibiting bacterial growth.

SOD is an active substance in organisms that can eliminate harmful substances produced during metabolism. Figure 7C shows that HBA could inhibit SOD activity. The HBA concentration was negatively correlated with SOD activity. When the HBA concentration was 250 μg/mL, the SOD activity was the lowest at 1.94 U/mg Prot, which was 0.06-fold that of the control (*p* < 0.05). This shows that HBA could disrupt antioxidant capacity in the bacteria, accelerate oxidation, and generate more harmful substances, thereby inhibiting bacterial growth.

GSH-PX is a scavenging enzyme that can scavenge in vivo hydrogen peroxide to protect cells from H_2_O_2_ toxicity and is an important enzyme in the defense system of an organism. Figure 7D shows that HBA inhibited GSH-PX activity. The HBA concentration was negatively correlated with GSH-PX activity. When the HBA concentration was 250 μg/mL, the GSH-PX activity was the lowest at 126.60 U/mg Prot, which was 0.59-fold that of the control (*p* < 0.05). This shows that HBA increases hydrogen peroxide and apoptosis.

### 2.8. Effects of HBA on Bacterial Cell Membrane Permeability

**Figure 8:**
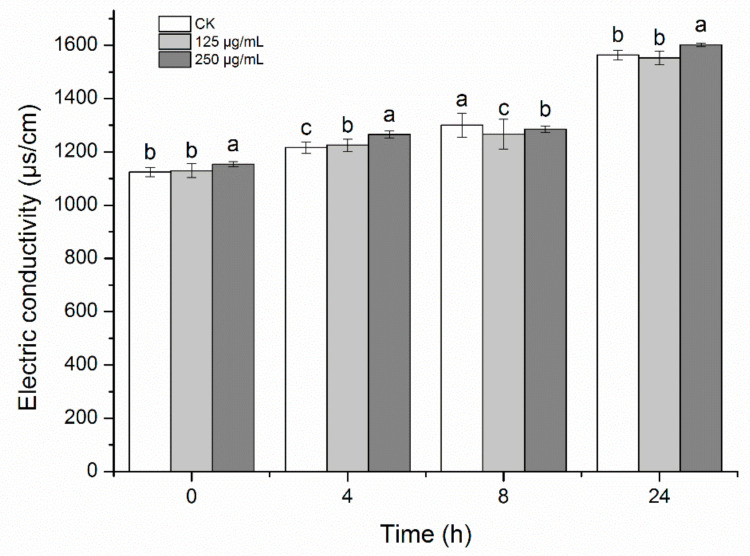
Effect of HBA on membrane permeability of *A. avenae* subsp. *citrulli*. Note: Columns followed by different letters indicate significantly different scores in the same phase according to Duncan’s multiple range tests at the *p* < 0.05 significance level.

Leakage of intracellular small molecules (such as Na+ and K+) will occur due to changes in membrane permeability in bacteria, thereby affecting conductivity. Conductivity in the HBA treatment group and control group increased with time, but conductivity in the HBA group was higher than in the control group (Figure 8). The higher the HBA concentration, the higher the conductivity. Conductivity was the highest when the HBA concentration was 250 μg/mL. However, conductivity in the low-concentration treatment group was slightly lower than the control at 8 and 24 h, which may be due to new chemical reactions of the leaked ions, resulting in decreased conductivity. In summary, HBA could increase bacterial cell membrane permeability and the leakage of intracellular substances, causing cell death.

### 2.9. Effects of HBA on Bacterial Cell Membrane Integrity

**Figure 9:**
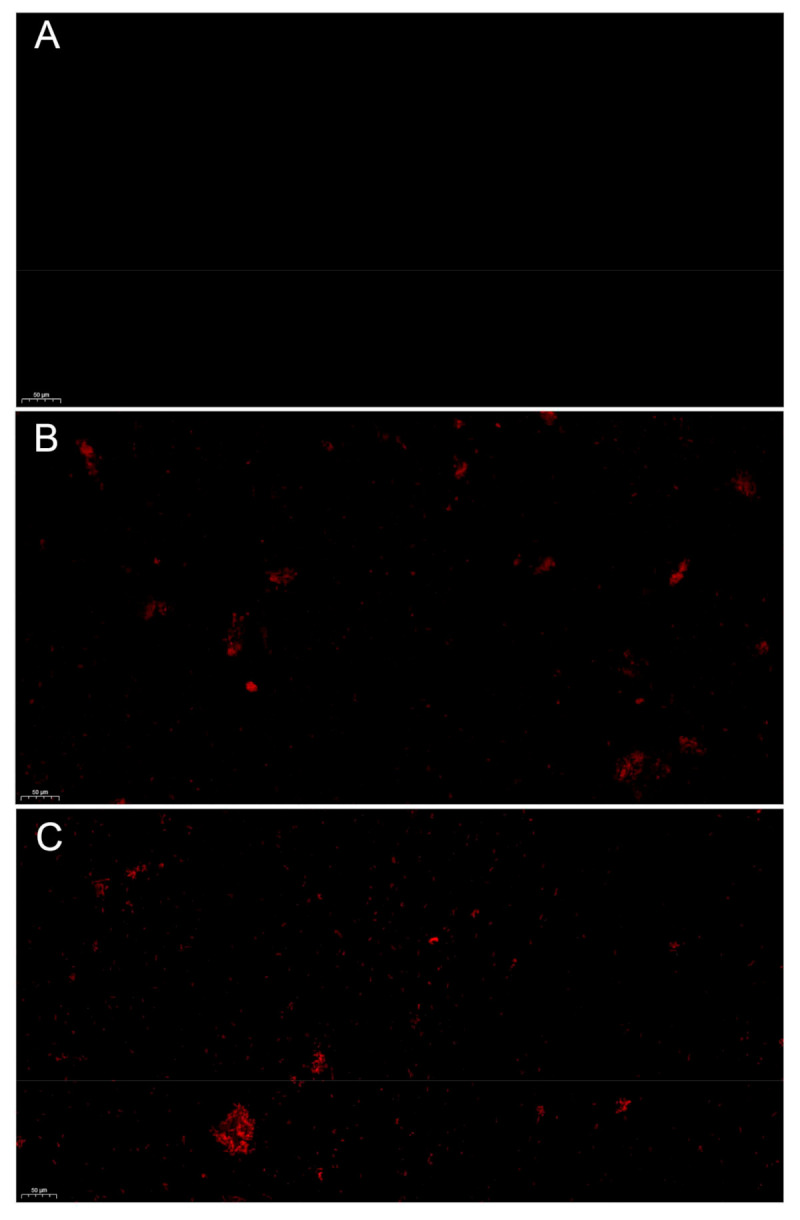
Cell membrane integrity of HBA against *A. avenae* subsp. *citrulli*. Note: (**A**): CK; (**B**): 125 μg/mL; (**C**): 250 μg/mL.

The fluorescent die propidium iodide (PI) is a dye that stains DNA. If PI can permeate the cell membrane and bind to DNA, the cell membrane integrity has been disrupted. If not, cell membrane integrity has not been affected. HBA could affect cell membrane integrity. PI staining was not red in the untreated bacteria (Figure 9A), but it was red in the HBA-treated bacteria (Figure 9B). The HBA concentration was positively correlated with PI staining intensity: the higher the concentration, the better the PI staining result. PI staining was not red in the untreated bacteria (Figure 9C). This shows that HBA could disrupt cell membrane integrity.

### 2.10. HBA on Pyruvate Level in A. avenae subsp. citrulli

**Figure 10:**
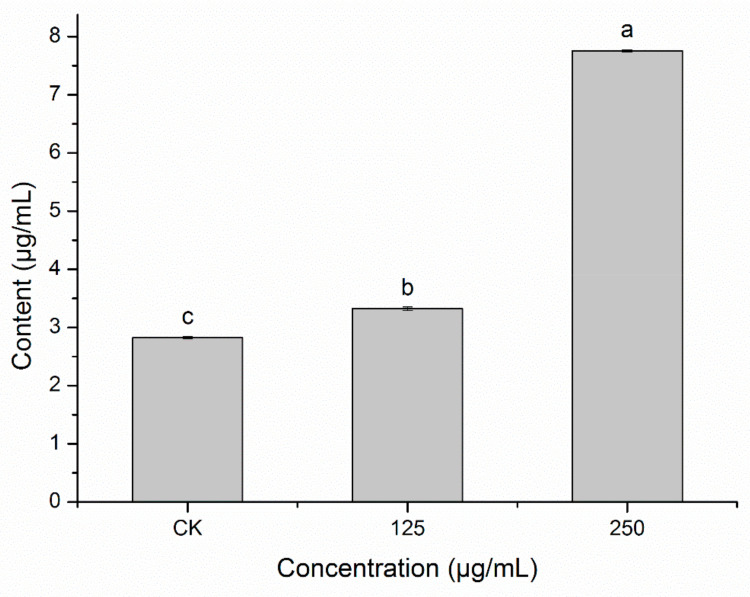
Pyruvic acid content effect of HBA against *A. avenae* subsp. *citrulli*. Note: Columns followed by different letters indicate significantly different scores in the same phase according to Duncan’s multiple range tests at the *p* < 0.05 significance level.

Pyruvate is an important intermediate in the tricarboxylic acid cycle and its level can effectively reflect changes in in vivo metabolic pathways. HBA significantly affected the normal metabolism of pyruvate in bacteria. The pyruvate level in the bacteria was significantly higher after HBA treatment compared with the control, and this difference was significant (*p* < 0.05). The higher the HBA concentration, the greater the pyruvate level in the bacteria (Figure 10). This shows that HBA affects the tricarboxylic acid cycle in bacteria and accelerates pyruvate metabolism.

### 2.11. Binding Model of HBA and Targets 

SWISS-MODEL was used for model construction. The results showed that MDH, NADH oxidase, SOD, and GSH-PX had the best homology with 1 mld, 5 jwa, 1 ptz, and 6 rjn, respectively, with values of 52.58%, 31.22%, 55.33%, and 54.57%, respectively. The GMQE was 0.69 and QMEAN was −0.77 for the MDH model; the GMQE was 0.33 and QMEAN was −3.85 for the NADH oxidase model; the GMQE was 0.14 and QMEAN was −0.97 for the SOD model; and the GMQE was 0.59 and QMEAN was −2.80 for the GSH-PX model, showing that these four models were generally reasonable. Verify-3D rationality mainly measures whether the compatibility of the number of amino acids and 3D-1D is ≥0.2. The MDH, NADH oxidase, SOD, GSH-PX target model Verify-3D evaluation results were 95.70%, 87.73%, 99.04%, and 72.96%, respectively, showing that the MDH, NADH oxidase, SOD, and GSH-PX target models were generally reasonable and basically satisfied subsequent molecular docking studies.

**Figure 11:**
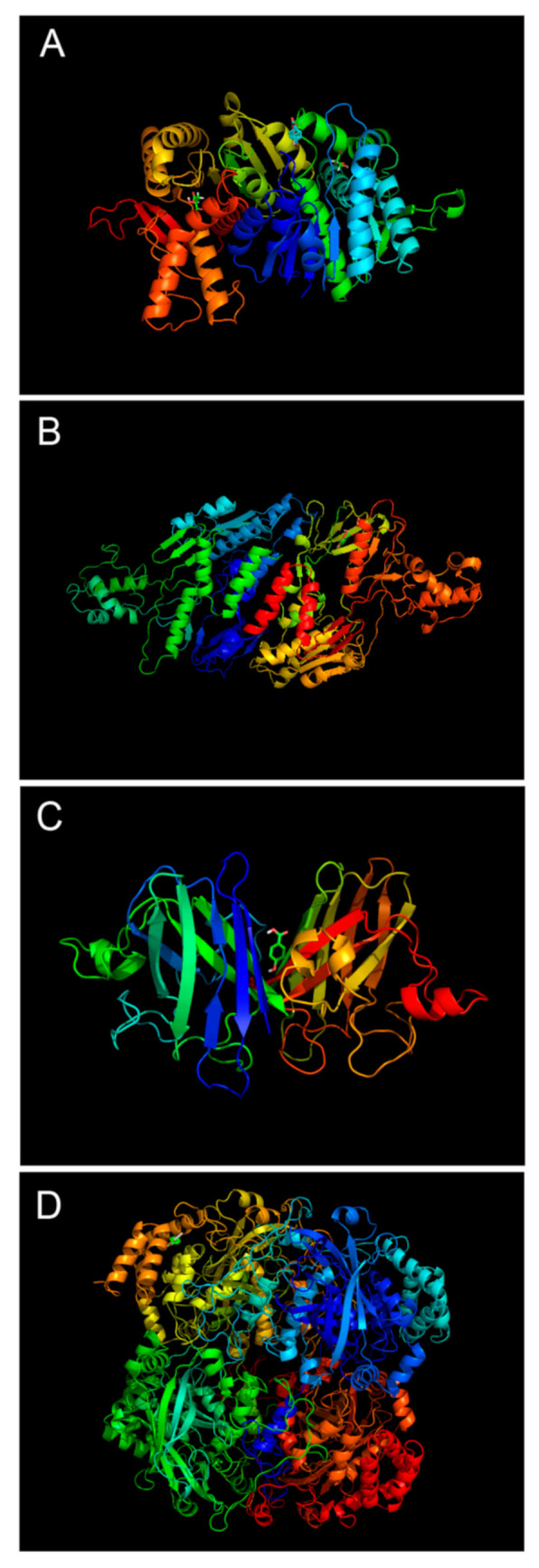
The theoretical binding mode between HBA and target enzymes and the result were shown by PyMoL 1.7.6. Notes: (**A**): binding complex of HBA and MDH; (**B**): binding complex of HBA and NADH oxidase; (**C**): binding complex of HBA and SOD; (**D**): binding complex of HBA and GSH-PX.

Molecular docking was used to study the HBA binding to the MDH, NADH oxidase, SOD, and GSH-PX target models. The results showed that HBA could bind to the MDH, NADH oxidase, SOD, and GSH-PX target models, and the binding free energy was −4.54, −4.11, −5.09, −3.73, respectively, of which HBA showed the best binding result with SOD (Figure 11).

HBA formed hydrogen bonds with Lys107 and Arg37 in MDH; Arg287 and Ile296 in NADH oxidase; Lys589 in SOD; and Arg620 in GSH-PX. In addition, van der Waals forces, electrostatic interactions, and hydrophobic interactions may be present between HBA and amino acid residues in other target models.

**Figure 12:**
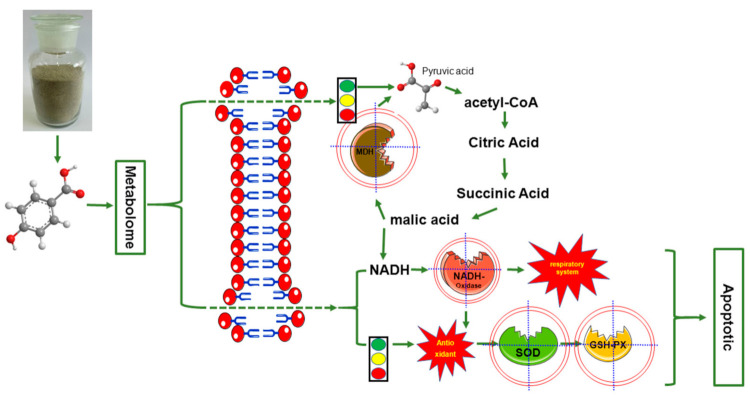
Inhibition molecular mechanism model of HBA against *A. avenae* subsp. *citrulli*.

In short, the molecular mechanism of HBA inhibiting *A. avenae* subsp. *citrulli* may be as follows: HBA damaged the permeability and integrity of cell membrane, entered the cytoplasm to affect the TCA cycle, destroyed MDH activity, blocked the dehydrogenation of related enzymes, reduced the number of NADH, reduced the activity of NADH oxidase due to the lack of substrate, and abnormal electron transfer in the respiratory chain, which further reduced the activities of SOD and GSH-PX in the antioxidant system, and led to cell apoptosis (Figure 12).

## 3. Discussion

The diversity of plants in nature provides an important material basis for biological pesticide research. Identifying lead compounds with bactericidal effects in plant materials is an integral component of biological pesticide development [23]. Our group previously found that *A. tricolor* is an important precursor material for producing biological pesticide, but its bacteriostatic components and mechanisms were unclear. In this study, we employed column chromatography, TLC, mass spectrometry, and NMR to isolate and identify two active ingredients in *A. tricolor* that inhibit *A. avenae* subsp. *citrulli*, of which HBA had the best bacteriostatic effects, with a zone of inhibition of 2.29 cm and MIC of 125 μg/mL. BPC was first isolated from *A. tricolor* but exhibited weak bacteriostatic activity. This is the first study to prove that HBA can inhibit *A. avenae* subsp. *citrulli*. Studies have found that HBA has applications as a food preservative, pro-apoptosis agent, and liquid crystal material [24,25,26]. This study identified new functions for HBA. Through a comparison with the toxicity of a commercially available bactericidal compound, we found that the bactericidal effect of HBA was lower than in Zhu et al. but significantly higher than that reported by Chen et al. and Jonkers et al. [27,28,29]. This shows that HBS is the best candidate compound for the prevention and treatment of *A. avenae* subsp. *citrulli*. Based on the aforementioned results, SEM was used to examine the effects of HBA on bacterial ultrastructure. The results showed that HBA resulted in bacterial distortion and deformation, uneven surfaces, and even disintegration. This is generally consistent with many previous studies [30]. Some lead compounds affect bacterial morphology by inducing shriveling, rupture, fragmentation, disintegration, and deformation, which inhibit bacterial growth and achieve bacteriostatic effects. This shows that HBA has application as a potential bactericidal agent.

Metabolomics was used to study the effects of HBA on metabolites in bacteria. The results showed that there were significant changes in bacterial metabolites after HBA treatment, indicating that HBA can result in metabolic disturbance in bacteria. The study of Tremaroli et al. proved that metals can result in metabolic changes in bacteria [31]. This shows that compounds can cause metabolite changes in bacteria, which is an important pathway for discovering new effector targets [32]. HBA mainly affected metabolites such as strigolactones, organic acids, fatty acids, benzene and substituted benzene derivatives, amino acids, alcohols, amines, and other metabolites. These metabolites play important roles in life activities in cells.

Amino acids are the precursors of proteins and nucleotides and participate in in vivo protein and nucleotide synthesis [33,34]. The results of this study showed that significant changes occurred in many amino acids, of which the downregulation of S-adenosyl-L-methionine (MEDP1280) was the most critical, as it affected the biosynthesis of amino acids, monobactam biosynthesis, arginine and proline metabolism, and the sulfur relay system. The results of previous studies proved that S-adenosyl-L-methionine has methyltransferase, sulfotransferase, and aminopropyl transferase effects and is a precursor for cysteine, taurine, glutathione, and coenzyme A [35]. The results of this study were generally consistent with the amino acid results of previous studies, proving that amino acids are an important bactericidal target and suggesting that amino acids may be HBA targets [36]. However, whether the reason for the downregulation of these substances was due to decreased synthase activity or the consumption of downstream metabolites remains to be determined. Of course, downregulation of these substances can stop the downstream synthesis of cysteine and glutathione, thereby causing bacterial death. This shows that these substances may be potential targets of HBA.

Organic acids in bacteria can maintain a neutral pH to ensure that enzymes can work normally [37]. The results of this study showed that 2,5-dihydroxy benzoic acid (MEDN0089) was upregulated several-fold higher than 2-amino-3-phosphonopropionic acid (MW0104504), which may cause organic acid levels to increase more than decrease, showing that elevated intracellular pH can be used to resist HBA stress. However, pH elevation causes dysregulated enzyme function and accelerated cell senescence. Second, the subsequent enzymatic activity measurements in this study also confirmed this point. Antioxidant enzyme activity decreased after HBA treatment, particularly GSH-PX. This directly resulted in intracellular reactive oxygen species accumulation. Excess reactive oxygen species will attach unsaturated fatty acids to the cell membrane, resulting in cell disruption, protein disturbances, and the production of aldehydes, further damaging cells. Messenger metabolites also play important intracellular roles. The results of this study showed that HBA can downregulate Bis(1-inositol)-3,1′-phosphate 1-phosphate (MEDN1224), which usually acts as a messenger and has important regulatory effects in cells. This suggests that HBA could block signaling molecule activity and intracellular signal transduction to impede bacterial growth, and these may be the potential targets of HBA. In addition, intracellular phenolic metabolites are mostly related to stress resistance. The results of this study showed that phenol (MEDL01916) and 2-(4-hydroxyphenyl) ethanol (MEDN0554) were upregulated, suggesting that these may be related to stress resistance. Fatty acids can provide energy, and saturated fatty acids mostly accelerate apoptosis, while unsaturated fatty acids do not have this function [38]. The results of this study showed that intracellular palmitoleic acid (MEDN0380) and tetracosanoic acid (MW0169908) were upregulated after HBA treatment. This was opposite to the results of Wang et al., who found that intracellular fatty acids were downregulated when bacteria were stimulated in a space environment [39]. This shows that changes in fatty acid metabolism differ under different external conditions. The reason for this may be that the effects of different external stimuli on signaling substances vary, thereby affecting signal transduction and resulting in differences in fatty acid metabolism.

The cell membrane can maintain cell morphology and has protective effects on the cell. At the same time, the cell membrane must take in essential nutrients and oxygen from the surrounding environment and expel metabolites. The cell membrane has selective permeability and can exclude harmful substances or macromolecules from entering the cell to maintain intracellular homeostasis.

Therefore, cell membrane integrity affects the life activities of the cell. The results of this study showed that HBA can damage cell membrane permeability and result in the leakage of intracellular substances. The PI staining results also showed that HBA could disrupt cell membrane integrity. This indicates that the cell membrane may be a potential effector target of HBA. This is generally consistent with previous reports that bactericidal compounds act on the cell membrane and confirms that HBA has basic bactericidal characteristics and can be used to develop a bactericide [40]. However, how HBA disrupts cell membrane permeability and integrity, such as what substances are first bound by HBA, what responses are triggered after binding, and what effects occur after these responses, requires further analysis.

Respiration is an important means for organisms to perform life activities and is characterized by intracellular oxidative metabolism. The inhibition of glucose metabolism will block organism growth and proliferation and may even lead to death [41]. The results of this study showed that HBA can increase the pyruvate level in bacteria, indicating that the TCA cycle is disrupted after HBA treatment. To resist this disruption, pyruvate levels increase to compensate for energy deficiency. However, further studies are required to understand when pyruvate levels start to increase, peak, and end, and what the subsequent cascade effects after HBA treatment are. Second, HBA can also decrease the activities of MDH and NADH oxidase, proving that HBA inhibits bacterial respiration to decrease energy and slow down metabolism. However, further studies are required to determine if HBA influences other respiratory enzymes, whether inhibition is simultaneous or successive, and what factors control these enzymes.

Antioxidant enzymes are biological catalysts produced in vivo that can slow down oxidation speed. Antioxidant enzymes is an umbrella term for SOD, catalase, etc. Once peroxides are produced, these enzymes immediately perform their functions and use oxidoreduction to convert peroxides to harmless or less toxic substances [42]. Studies have found that HBA decreases SOD and GSH-PX activities, suggesting that HBA acts on the antioxidant enzyme system. An impaired antioxidant system in bacteria causes them to be susceptible to death.

Molecular docking was used to study the binding between HBA and respiratory and antioxidant enzymes. The results showed that HBA could bind to MDH, NADH oxidase, SOD, and GSH-PX, of which SOD exhibited the best binding, showing that SOD may be an effector target of HBA. However, binding between HBA and these enzymes was not optimal, and molecular dynamics should be used in future studies to determine optimal binding methods.

*Amaranthus tricolor* has diverse and complex components. In this study, column chromatography was used for isolation and purification, and most of the substances obtained were acidic substances. However, previous studies reported that other types of bacteriostatic compounds are present in *A. tricolor*. Therefore, the isolation and purification methods could be further improved to obtain more valuable natural active compounds. Second, this study only performed indoor toxicity evaluation. In the future, field prevention and treatment studies could be considered to further validate the bacteriostatic activity of HBA, which will help guide subsequent studies. Third, in-depth studies are required to determine if HBA will affect the pathogenic secretory systems (type II and III) in the bacteria, exopolysaccharide (EPS), toxins, growth regulators, and flagella [43,44,45,46]. Lastly, future studies are required to determine if HBA has regulatory effects on the cell wall, nucleic acid, and protein synthesis and whether there are new effector targets.

## 4. Materials and Methods

### 4.1. Test Plants

The *A. tricolor* plants were cultivated at the Liuyang Research Base of Hunan Agricultural University for 2 months. Mature *A. tricolor* plants (16–24 cm in height) were harvested and their leaves were collected, washed, and dried until the leaf moisture content was controlled at <5%. An XY-200 leaf pulverizer (Zhejiang Yongkang Songqing Hardware Factory) was used to powder the leaves, which was referred to as *A. tricolor* powder (A). The powder was stored dry at room temperature.

### 4.2. Preparation of Culture Media

Beef extract peptone culture medium (NA) consisting of 5.0 g beef extract, 10.0 g peptone, 5.0 g NaCl, 40.0 g agar, and 1000 mL distilled water at pH 7.0–7.2 was used. The above materials were heated and dissolved in 1000 mL distilled water. Water loss was topped up. After the substances had completely dissolved, agar was added and stirred until complete dissolution. The pH was adjusted to 7.0–7.2. The medium was aliquoted into 250 mL-conical flasks and autoclaved at 121 °C for 20 min before cooling [47].

### 4.3. Bacterial Strain Activation

The *A. avenae* subsp. *citrulli* was removed from the freezer and thawed to room temperature. An inoculating loop was used to select bacteria for inoculation onto fresh NA culture medium. This step was repeated 2–3 times. The bacteria were cultured continuously for 2–3 generations at 28–30 °C, and bacteria with good activity were selected.

### 4.4. Preparation of A. tricolor Ester Extract

Five volumes of ethyl acetate were used to extract 6.5 kg of *A. tricolor* powder (A) for 6 h with shaking (rotation speed: 150 r/min, temperature: 26 ± 1 °C). After leaving to stand for 24 h, the extract was filtered, and the same volume of ethyl acetate was used for crude extraction of the residue for 24 h before filtration. The filtrates were combined and subjected to rotary concentration at 40 °C to obtain a paste. The paste was weighed, named *A. tricolor* black crude extract (B), and stored at 4 °C. The above filtrates were also eluted by carbon column chromatography to remove the pigment in the crude extract. The chromatography column was filled with the activated carbon powder and diatomite at a 2:1 ratio, and the eluant was ethyl acetate. The colorless chromatography liquid was finally rotary concentrated at 40 °C to obtain a colorless paste, named *A. tricolor* colorless extract and stored at 4 °C.

### 4.5. Measurement of A. tricolor Ester Extract Activity

Activated *A. avenae* subsp. *citrulli* was diluted with sterile water to prepare a 1 × 10^8^ cfu/mL bacterial suspension. Three milliliters of suspension and 27 mL of NA culture medium were fully mixed and poured onto a sterile Petri dish. After the culture medium had completely solidified, a hole punch was used to punch wells, and 200 μL of *A. tricolor* black crude extract (B) was added to the wells. An untreated plate was used as a control. Each treatment was repeated in triplicate. The Petri dishes were cultured at 28–30 °C. After bacterial growth in the blank control plate was confluent, the zone of inhibition was measured. In addition, the minimum inhibitory concentration (MIC) was measured as described previously [48]: The final concentration of novel fungicide HBA, BFC and ZSM were 0.5 times gradient decrement from left to right using the double dilution method of a 96-well plate with each treatment in duplicate. The first well contained 500.00 μg/mL and the second well contained 250.00 μg/mL, etc. The ZSM was used as a positive control, and the untreated wells were used as a negative control. The Petri dishes were cultured at 28–30 °C for 24 h, and the lowest concentration with no bacterial growth was considered to be the MIC for *A. tricolor* pure product.

### 4.6. Silica Gel Column Chromatography

The *A. tricolor* black crude extract (B) was dissolved in 500 mL ethyl acetate, and the solution was slowly injected into an activated carbon column so that the crude extract slowly passed through the carbon powder column to remove pigments in the extract. The column was repeatedly washed with ethyl acetate until the eluate was colorless. All of the filtrates were collected and subjected to rotary evaporation at 40 °C until a paste was obtained, which was named *A. tricolor* yellow paste (C). A silica gel column (glass column diameter and height of 6.5 × 60 cm, column volume: 200 mL) was used for isolation. The sample was loaded using the wet method. Thirty-four grams of *A. tricolor* yellow paste (C) was loaded, and gradient elution was carried out using trichloromethane:methanol = 1:0, 9:1, 4:1, 7:3, 3:2, 0:1 (V/V). Collection was performed once every 250 mL, and TLC was used to test every fraction. Fractions with the same components were combined and subjected to vacuum concentration before the bacteriostatic activity was measured. The bacteriostatic fractions were named as *A. tricolor* bacteriostatic fraction (D) and stored at −20 °C.

### 4.7. ODS-C18 Reverse-Phase Chromatography

The dry method was used to pack ODS-C18 filler into the column. The filler occupied 98% of the column, and methanol was used to wet the column. A medium pressure constant current pump was used to slowly add methanol into the reverse-phase column, and the flow rate was controlled at 2.22 mL/min. After no bubbles appeared and the filler had been completely immersed in methanol, the flow rate was increased to 4.44 mL/min and washing was continued for 2 h. Methanol (30%) was used to wash the column for 6 h at 2.22 mL/min. Then, 3–5 g of *A. tricolor* bacteriostatic fraction (D) was mixed evenly with an appropriate amount of ODS-C18 silica gel. The solvent was completely evaporated for loading. The silica gel was added to the reverse-phase column and the elution sequence was performed: 30–100% methanol for gradient elution at a flow rate of 4.44 mL/min. Every elution was washed with 1000 mL of elution solution and collection was performed every 100 mL. TLC was used to test every fraction. Fractions with the same components were combined and subjected to vacuum concentration before the bacteriostatic activity was measured. Fractions with bacteriostatic activity were named *A. tricolor* bacteriostatic fraction (E) and stored at −20 °C.

### 4.8. Sephadex LH−20 Gel Filtration Chromatography

Three-hundred grams of Sephadex LH-20 gel was weighed in a beaker, and 500 mL of pure methanol was added to soak the gel overnight. The column was loaded using the wet method. The treated gel was stirred until the solution became turbid. A glass rod was used to place the soaked gel onto a 1.5 × 200 cm column. The entire gel was completely transferred to the column, and pure methanol was used for flushing. After continuous flushing for 2 h, the column piston was closed. The solvent level was 10 cm from the gel. The column was left to stand overnight. The bottom piston was removed to allow liquid from the gel filtration column to flow out. The solvent level in the solid phase was controlled at 0.5 cm from the gel level and the column piston was closed. The *A. tricolor* bacteriostatic fraction (E) was dissolved in 1–2 mL of pure methanol, and a long dropper was used to slowly add the dissolved sample onto the gel filtration column. After the sample was completely transferred to the column, the piston was opened, and the sample solution was allowed to completely immerse in the solid phase at a flow rate of 0.5 mL/min. After that, methanol was used for elution. Collection was performed every 5 mL. Each tube of eluate underwent TLC analysis, and identical fractions were combined and subjected to vacuum concentration before the bacteriostatic activity was measured. Highly pure compounds 1 and 2 were obtained.

### 4.9. Structural Identification of Compounds

The electrospray ionization−mass spectrometry (ESI−MS) working conditions were as follows: the MS column was 2.6 mm × 250 mm, 2 μm; the detection wavelength was 254 nm; the flow rate was 0.5 mL/min; the electrospray operating voltage was 4.02 kV; the operating current was 0.39 µA; the capillary temperature was 275 °C; and the drying gas (N_2_) flow rate was 19.92 L/min.

The NMR operating conditions were as follows: the sample was dissolved in deuterated chloroform (CDC13), or deuterated methanol (CD4O) and scanning was performed on a Bruker Avance 500 MHz spectrometer. The hydrogen spectrum and carbon spectrum were obtained, and the test temperature was 297.3 K. Tetramethylsilane (TMS) was used as an internal standard for both the hydrogen and carbon spectra. The test spectrum width of the 1 H NMR and 13 C NMR spectra was 10,330.578 and 22,058.824 Hz, respectively.

### 4.10. Effects of HBA on Bacterial Ultrastructure Using Scanning Electron Microscopy (SEM)

A small volume of methanol and compound 1 was used to prepare 125 μg/mL bacteria-containing solution, with a bacteria-free solution used as a control. The solutions were cultured with shaking at 180 r/min for 24 h. After that, the solutions were centrifuged at 10,000 r/min for 5 min. The supernatant was discarded, and 2 g of pellet was collected. After processing with the electron microscopy fixing solution (Reagent number: G1102, Wuhan Servicebio Technology Co., Ltd., Wuhan, China), electron microscopy observation of bacterial changes was performed as described [49].

### 4.11. Bacterial Metabolome

The method in 2.10 was used to process the bacteria, and the processing groups were named TM1, TM2, and TM3 and the control groups were named CK1, CK2, and CK3. Around 2 g of pellet was collected and frozen in liquid nitrogen. The samples were stored in a −80 °C freezer before sending to a biotechnology company for metabolomics testing. For extraction, the samples were removed from the −80 °C freezer and thawed on ice. Two-hundred microliters of ultrapure water extraction solution was added, and the samples were vortexed for 3 min until the cell suspension was uniform. When there was difficulty obtaining a uniform suspension, a pipette was used to mix the sample. One-hundred microliters of cell suspension was aspirated into the corresponding centrifuge tube, and 400 μL pure methanol internal standard extraction solution was added. Samples were vortexed for 1 min. Centrifuge tubes were snap-frozen in liquid nitrogen for 5 min and thawed on dry ice for 5 min before thawing on ice for 5 min. Samples were vortexed for 2 min to mix evenly before snap-freezing, thawing, and vortexing, which were repeated thrice. Samples were centrifuged at 4 °C and 12,000 r/min for 10 min. Three-hundred microliters of supernatant was aspirated to the corresponding numbered centrifuge tube and left to stand in a −20 °C freezer for 30 min. The tubes were recentrifuged at 4 °C and 12,000 r/min for 3 min. Two-hundred microliters of supernatant was aspirated to the sampling vial and used for UPLC-MS-MS analysis.

For the liquid chromatography analysis, the data acquisition instrument system mainly included an Ultra-Performance Liquid Chromatograph (UPLC) (ExionLC AD, https://sciex.com.cn, accessed on 15 October 2021) and tandem mass spectrometry (MS/MS) (QTRAP^®^, https://sciex.com, accessed on 17 October 2021).

The chromatography column was a Waters ACQUITY UPLC HSS T3 C18 1.8 µm, 2.1 mm × 100 mm; mobile phase A was ultrapure water (0.1% formic acid) and mobile phase B was acetonitrile (0.1% formic acid); the elution gradient was 0 min water/acetonitrile (95:5 V/V), 11.0 min: 10:90 V/V, 12.0 min: 10:90 V/V, 12.1 min: 95:5 V/V, 14.0 min: 95:5 V/V; the flow rate was 0.4 mL/min; the column temperature was 40 °C; and the sample volume was 2 μL. The T3 method was used for the generation of mass spectra with an electrospray ionization (ESI) temperature of 500 °C; mass spectrometry voltage of 5500 V (positive) and −4500 V (negative); ion source gas I (GS I) of 55 psi; gas II (GS II) of 60 psi; and curtain gas (CUR) of 25 psi; and the collision-activated dissociation (CAD) parameter was set as high. In the triple quadrupole (Qtrap), every ion pair was scanned and detected based on the optimized declustering potential (DP) and collision energy (CE) [50]. Analyst1.6.3 software was used to process the mass spectrometry data.

### 4.12. Bacterial Cell Membrane Permeability

Based on the experimental method of Wei [51], the density of the *A. avenae* subsp. *citrulli* suspension in the logarithmic growth phase was adjusted to OD600 = 0.5, and HBA was added to a final concentration of 125 and 250 μg/mL. Ultrapure water was used as a control. After treatment for 0, 4, 8, and 24 h, 5 mL of culture medium was collected and centrifuged at 6000 r for 10 min. The supernatant was diluted 20-fold with ultrapure water, and a conductivity meter was used to measure conductivity. Every treatment was performed in triplicate.

### 4.13. Bacterial Cell Membrane Integrity

The bacteria were processed according to the method in 4.10, and PI staining was performed according to Chavan and Tupe [52] to observe the effects of compound 1 on bacterial cell membrane integrity.

### 4.14. Effects of HBA on Bacterial Pyruvate Level

The bacteria were processed according to the method in 4.10, and the method of Huang et al. [53] was used to measure the pyruvate level.

### 4.15. Effects of HBA on Bacterial Enzymatic Activity

The logarithmic growth phase bacterial suspension was adjusted to OD_600_ = 0.5, and HBA was added to a final concentration of 125 and 250 μg/mL. The bacteria-free suspension was used as a control. Samples were cultured for 24 h at 28–30 °C. Two milliliters of the solution was collected and centrifuged at 4 °C 600 rpm for 5 min. The pellet was dissolved in phosphate buffer and sonicated (power: 100%, sonication duration: 3 s, interval: 10 s, repeated 30 times). The solution was centrifuged at 4 °C and 11,000 rpm for 10 min. The supernatant was then collected, and the concentration of the crude enzyme was measured. Coomassie blue was used to measure the concentration of the crude enzyme. Enzyme activity was measured according to the malate dehydrogenase A021-2-1 (MDH, Nanjing Jiancheng Bioengineering Institute), NADH oxidase A020-1-1 (NADH, Nanjing Jiancheng Bioengineering Institute), superoxide dismutase A001-1 (SOD, Nanjing Jiancheng Bioengineering Institute), and glutathione peroxidase A005-1 (GSH-PX, Nanjing Jiancheng Bioengineering Institute) assay kits. Each treatment was performed three times.

### 4.16. Molecular Docking

The amino acid sequences of MDH A021-2-1 (Nanjing Jiancheng Bioengineering Institute), NADH oxidase A020-1-1 (NADH, Nanjing Jiancheng Bioengineering Institute), superoxide dismutase A001-1 (SOD, Nanjing Jiancheng Bioengineering Institute), and glutathione peroxidase A005-1 (GSH-PX, Nanjing Jiancheng Bioengineering Institute) were obtained from the GenBank database, and Blast was used to search for homology models on the website. The SWISS-MODEL (http://swissmodel.Expasy.org, accessed on 6 August 2021) program was used to construct the molecular model of the target protein. The structure of HBA was plotted using ChemBioDraw Ultra 12.0 (PerkinElmer Co., Ltd., MA, USA) and converted into a three-dimensional structure. At the same time, optimization was performed. GMQE, QMEAN, and Verify-3D were used to evaluate the quality of the protein model. Autodock v4.2 (Olson Laboratory of Scripps Institute, La Jolla, CA, USA) was used for molecular docking, and the results were analyzed using Pymol v1.7.6.

### 4.17. Data Analysis

The ^1^H-NMR and ^13^C-NMR data were processed using the software (MestReNov v14.0, Mestrelab Research S.L, San Diego, Spain) provided with the instrument. A completely random design was employed for the experiment. Excel v2016 was used for statistical analysis of the data. All of the data represent the mean and standard deviation of triplicates. Origin software was used for graph plotting. Duncan’s new multiple range test in DPS v 6.55 was used for significant difference analysis.

## 5. Conclusions

This study was the first to develop a method for isolating and purifying *A. tricolor* substances that inhibit *A. avenae* subsp. *citrulli* viability. Two components were identified, of which HBA had the highest bacteriostatic activity and potential use in the development of a biological pesticide. Further analysis found that HBA could cause bacteria to become shriveled, distorted, and deformed and cause uneven bacterial surfaces. Metabolomics analysis found that 70 differentially expressed metabolites that respond to HBA were detected, of which 9 were downregulated (which may be potential effector targets) and 61 were upregulated (which may be related to drug resistance). The differentially expressed metabolites were mainly strigolactones, organic acids and derivatives, fatty acids, benzene and substituted benzene derivatives, amino acids and associated metabolites, alcohols and amines, and other metabolites. Among all of the downregulated metabolites, S-adenosyl-L-methionine (MEDP1280) was the most critical as it participates in many physiological and biochemical processes. The KEGG enrichment analysis showed that the differentially expressed metabolites mainly participated in tyrosine metabolism, biosynthesis of amino acids, and cysteine and methionine metabolism. HBA can disrupt cell membrane permeability and integrity. The pyruvate level increased, while the activities of MDA, NADH oxidase, SOD, and GSH-PX decreased after HBA treatment. The inverse molecular docking study results showed that HBA could dock to MDA and NADH oxidase, SOD, and GSH-PH, of which the docking result was the best for SOD, suggesting that SOD may be the best effector target of HBA.

## Figures and Tables

**Table 1:** Inhibition effect of three fungicides against *A. avenae* subsp. *citrulli*.

Compound	Inhibition Zone Diameter (cm)
	Average Value
HBA	2.29 ± 0.05 ^a^
BFC	0.54 ± 0.02 ^b^
Zhongshengmycin (positive control)	2.23 ± 0.01 ^a^

Note: Different lowercase letters indicate significant differences at the *p* < 0.05 level.

**Table 2:** Significantly different metabolites putatively identified by UPLC-MS/MS.

Number	Index	Formula	Compounds	Log2FC	Type
1	MW0152046	C_22_H_27_NO_5_	Kreysigine	12.87	up
2	MW0169908	C_24_H_48_O_2_	Tetracosanoic acid	12.52	up
3	MEDN1492	C_8_H_8_O_3_	(R)-(-)-Mandelic acid	11.96	up
4	MEDN0343	C_7_H_10_O_5_	Shikimic Acid	11.61	up
5	MEDL01916	C_6_H_6_O	Phenol	5.68	up
6	MEDN1167	C_7_H_6_O_3_	4-Hydroxybenzoic Acid	5.49	up
7	MEDN0554	C_8_H_10_O_2_	2-(4-Hydroxyphenyl)ethanol	5.47	up
8	MW0155866	C_13_H_19_N_5_O_4_	Pro Gly His	5.28	up
9	MEDN0089	C_7_H_6_O_4_	2,5-Dihydroxy Benzoic Acid	4.98	up
10	MEDN1224	C_12_H_24_O_17_P_2_	Bis(1-inositol)-3,1′-phosphate 1-phosphate	−1.66	down
11	MEDN0481	C_7_H_6_O_4_	2,4-Dihydroxy Benzoic Acid	4.98	up
12	MEDN1322	C_7_H_6_O_4_	2,3-Dihydroxybenzoic acid	4.98	up
13	MEDN1701	C_7_H_6_O_4_	3,5-Dihydroxybenzoic acid	4.98	up
14	MW0009845	C_16_H_22_ClN_3_O	Tebuconazole	4.52	up
15	MEDL00416	C_12_H_24_O_3_	3-Hydroxydodecanoic acid	4.31	up
16	MW0013514	C_10_H_20_O_3_	2-Hydroxydecanoate	4.20	up
17	MEDP1280	C_15_H_22_N_6_O_5_S	S-Adenosyl-L-Methionine	−1.09	down
18	MW0105037	C_10_H_20_O_3_	3-Hydroxycapric acid	4.14	up
19	MEDP1464	C_13_H_13_N_3_	1,3-Diphenylguanidine	−2.27	down
20	MEDN1604	C_14_H_28_O_3_	3-hydroxy-tetradecanoic acid	3.47	up
21	MW0015365	C_16_H_26_O_3_	8R-Hydroxy-4Z,6E,10Z-hexadecatrienoic acid	3.42	up
22	MW0111229	C_18_H_39_N	n-Octadecylamine	3.00	up
23	MW0122796	C_18_H_13_ClFN_3_O	Alpha-hydroxymidazolam	2.94	up
24	MW0141607	C_15_H_24_N_2_O_2_	17-Hydroxylupanine	2.86	up
25	MW0145377	C_15_H_26_N_8_O_5_	Arg His Ser	2.81	up
26	MW0158148	C_25_H_28_N_4_O_6_	Trp Glu Phe	2.77	up
27	MW0141608	C_22_H_32_O_3_	17-keto-7(Z),10(Z),13(Z),15(E),19(Z)-Docosapentaenoic Acid	2.70	up
28	MW0006902	C_13_H_13_OP	Diphenylmethylphosphine oxide	2.70	up
29	MEDN0380	C_16_H_30_O_2_	FFA(16:1)	2.70	up
30	MW0063608	C_30_H_48_O_2_	Soyasapogenol C;Sapogenol C	2.38	up
31	MEDN0494	C_20_H_32_O_4_	8,15-Dihete	2.35	up
32	MW0061673	C_18_H_40_NO_6_P	Phytosphingosine-1-P	2.24	up
33	MW0161385	C_27_H_44_O	(1S)-3-[(Z)-2-[(1R,7aR)-7a-methyl-1-[(2R)-6-methylheptan-2-yl]-1,2,3,3a,6,7-hexahydroinden-4-yl]ethenyl]-4-methylcyclohex-3-en-1-ol	2.17	up
34	MW0011819	C_17_H_33_O_7_P	1-(9Z-tetradecenoyl)-glycero-3-phosphate	2.17	up
35	MW0009514	C_23_H_31_NO_2_	Proadifen	2.14	up
36	MW0168308	C_21_H_30_O_4_	[10]-Dehydrogingerdione	2.13	up
37	MW0150989	C_17_H_27_N_5_O_6_	His Leu Glu	2.13	up
38	MW0009714	C_25_H_37_NO_4_	Salmeterol	2.13	up
39	MEDP1032	C_7_H_6_O_2_	Salicylaldehyde	2.13	up
40	MW0104504	C_3_H_8_NO_5_P	2-Amino-3-phosphonopropionic acid	−1.82	down
41	MW0145031	C_22_H_47_NO_5_	Aminopentol; AP1	2.12	up
42	MW0105559	C_6_H_12_N_2_O_4_	Ala-Ser	−1.15	down
43	MW0016192	C_25_H_37_NO_4_	Bimatoprost	2.06	up
44	MW0007815	C_17_H_20_N_2_O	Michler’s ketone	1.80	up
45	MW0017120	C_26_H_34_O_6_	Cinobufagin	1.78	up
46	MW0012068	C_21_H_30_O_3_	11a-Hydroxyprogesterone	1.74	up
47	MW0132792	C_15_H_10_O_5_	3,7,4′-Trihydroxyflavone	−13.61	down
48	MW0158708	C_29_H_26_N_2_O_7_	TyrMe-Nap-OH	1.70	up
49	MW0144201	C_13_H_15_N_3_O_7_	Abu-Asn-OH	1.65	up
50	MW0139399	C_16_H_12_O_6_	Pratensein	1.56	up
51	MW0142458	C_17_H_34_O_3_	2-Methoxyhexadecanoic acid 2-Methoxyhexadecanoate	1.56	up
52	MW0123384	C_24_H_34_O_7_	Clerodin	1.53	up
53	MW0155978	C_16_H_27_N_3_O_4_	Pro Pro Ile	1.47	up
54	MW0157221	C_30_H_42_O_7_	Stigmatellin A; Stigmatellin	1.43	up
55	MW0144075	C_22_H_39_NO_5_	8-iso Prostaglandin F2 Ethanolamide	−8.13	down
56	MW0137461	C_20_H_22_O_3_	Avobenzone	1.37	up
57	MW0015339	C_20_H_34_O_5_	8-iso-13,14-dihydro-15-keto-PGF2a	1.36	up
58	MW0145074	C_25_H_29_NO_4_	Ancistrocladine	−11.81	down
59	MW0016257	C_22_H_40_O_2_	Butyl 9,12-octadecadienoate	1.30	up
60	MW0149681	C_22_H_40_N_8_O_8_	Gln Glu Ile Arg	−1.48	down
61	MW0015555	C_17_H_24_O_4_	Acetylvalerenolic acid	1.29	up
62	MW0116826	C_21_H_28_O_4_	11-nor-9-carboxy-Delta(9)-tetrahydrocannabinol	1.29	up
63	MW0005044	C_13_H_16_O_4_	4-hydroxy-2-methoxy-3-(3-methylbut-2-en-1-yl)benzoic acid	1.28	up
64	MEDP1685	C_18_H_39_NO_3_	Phytosphingosine	1.27	up
65	MW0012516	C_22_H_36_O_6_	16,16-Dimethyl-6-ketoprostaglandin E1	1.22	up
66	MW0143701	C_11_H_14_N_4_O_4_S	5′-S-Methyl-5′-thioinosine; 5′-Deoxy-5′-methylthioinosine; S-Methyl-5′-thioinosine	1.20	up
67	MEDN1516	C_15_H_10_O_3_	6-Hydroxyflavone (6-HF)	1.16	up
68	MW0013392	C_16_H_33_NO_2_	2-aminohexadecanoic acid	1.12	up
69	MW0143692	C_10_H_10_N4O_5_	5′-Oxoinosine; 5′-Dehydroinosine	1.10	up
70	MEDP1360	C_10_H_15_NO_6_	Mycosporine glycine	1.01	up

## Data Availability

The authors declare that the data supporting this study are available from the corresponding author on reasonable request.
